# The AD Biological Domains and Subdomains Expand the Multiomic Mapping into Data‐Driven Disease Endophenotypic Areas

**DOI:** 10.1002/alz70859_103831

**Published:** 2025-12-25

**Authors:** Jesse C Wiley, Gregory A. Cary, Laura M Heath, William L. Poehlman, Sruthi Ganesh, Allan I. Levey, Anna Greenwood, Gregory W Carter, Karina Leal

**Affiliations:** ^1^ Sage Bionetworks, Seattle, WA USA; ^2^ The Jackson Laboratory, Bar Harbor, ME USA; ^3^ Emory University, Atlanta, GA USA

## Abstract

**Background:**

The increasing requirement to harmonize multiomic datasets and apply interpretable biological mappings onto large scale analysis prompted us to incorporate the 19 Alzheimer’s Disease (AD) Biological Domains into the Target Enablement to Accelerate Therapy Development for AD (TREAT‐AD) bioinformatics pipeline. To further refine the specific biology implicated within each domain, we have broken the biological domains into a set of Alzheimer’s associated subdomains for more meaningful mapping of large datasets onto disease enriched biology.

**Method:**

There are two fundamental components to the TREAT‐AD bioinformatics pipeline: 1) the genome‐wide application of a gene‐centric risk score drawn from multiomic assessments; 2) the application of the risk score to drive enrichment into the endophenotypic and sub‐endophenotypic areas of disease associated biology. The latter part is performed in two steps, in which first the 19 biological domains are defined by an exhaustive set of Gene Ontology (GO) terms. Second, kappa‐networks are constructed from (a) the leading‐edge disease associated genes and (b) from the enriched GO terms. Within both network systems, the nodes represent the GO terms and the edges represent the conserved annotation of a set of genes between GO terms. This approach allows us to map “families” of biological processes that are linked to disease. Through filtration of the kappa‐value, the networks are deconstructed into subnetworks that represent subdomains, or subendophenotypes, associated with disease risk.

**Result:**

Employing the top‐down (GO‐term driven) and the bottom‐up (leading‐edge gene driven) methods of kappa‐network construction and subsequent fragmentation, we find a core set of biological subdomains associated with AD. Collectively, 115 subdomains are identified that are more specifically aligned with disease‐linked biology. For example, within mitochondrial metabolism (a leading downregulated domain) we observe electron transport chain (ETC) and tricarboxylic acid cycle (TCA cycle) as two of the 11 emergent subdomains.

**Conclusion:**

The AD Biological Domains and subdomain development will facilitate future alignment of multiomic datasets onto a common open‐source, open‐science platform for integrative analysis and hypothesis generation. Data‐driven standards for hypothesis generation may speed up the development of new therapeutic directions and drug target selection, opening new objective methods for translational advancement in AD.

